# The Pan-Immune-Inflammation Value is a new prognostic biomarker in metastatic colorectal cancer: results from a pooled-analysis of the *Valentino* and TRIBE first-line trials

**DOI:** 10.1038/s41416-020-0894-7

**Published:** 2020-05-19

**Authors:** Giovanni Fucà, Vincenzo Guarini, Carlotta Antoniotti, Federica Morano, Roberto Moretto, Salvatore Corallo, Federica Marmorino, Sara Lonardi, Lorenza Rimassa, Andrea Sartore-Bianchi, Beatrice Borelli, Marco Tampellini, Sara Bustreo, Matteo Claravezza, Alessandra Boccaccino, Roberto Murialdo, Alberto Zaniboni, Gianluca Tomasello, Fotios Loupakis, Vincenzo Adamo, Giuseppe Tonini, Enrico Cortesi, Filippo de Braud, Chiara Cremolini, Filippo Pietrantonio

**Affiliations:** 1 Medical Oncology Department, Fondazione IRCCS Istituto Nazionale dei Tumori di Milano, Milan, Italy; 2 Department of Translational Research and New Technologies in Medicine and Surgery, University of Pisa, Pisa, Italy; 3 Unit of Medical Oncology 2, Azienda Ospedaliero-Universitaria Pisana, Pisa, Italy; 4 Unit of Medical Oncology 1, Department of Clinical and Experimental Oncology, Istituto Oncologico Veneto, IRCCS, Padua, Italy; 5 Medical Oncology and Hematology Unit, Humanitas Cancer Center, Humanitas Clinical and Research Center-IRCCS, Rozzano, Italy; 6 Department of Biomedical Sciences, Humanitas University, Pieve Emanuele, Italy; 7 Niguarda Cancer Center, Grande Ospedale Metropolitano Niguarda, Milan, Italy; 8 Oncology and Hemato-oncology Department, University of Milan, Milan, Italy; 9 Department of Oncology, AOU San Luigi di Orbassano, University of Torino, Orbassano, Italy; 10 Colorectal Cancer Unit, Medical Oncology Division 1, AOU Città della Salute e della Scienza, Torino, Italy; 11 Medical Oncology Unit, Ente Ospedaliero Ospedali Galliera, Genoa, Italy; 12 Department of Internal Medicine, University of Genoa and IRCCS AOU San Martino-IST, Genoa, Italy; 13 Medical Oncology Unit, Fondazione Poliambulanza, Brescia, Italy; 14 Medical Oncology Unit, Azienda Socio-Sanitaria Territoriale (ASST) Ospedale di Cremona, Cremona, Italy; 15 Medical Oncology Unit, A.O. Papardo, Messina, Italy; 16 Department of Human Pathology, University of Messina, Messina, Italy; 17 Oncology Department, Policlinico Campus Bio-Medico di Roma, Rome, Italy; 18 Oncology Unit, Policlinico Umberto I, Rome, Italy

**Keywords:** Colorectal cancer, Prognostic markers

## Abstract

**Background:**

Immune-inflammatory biomarkers (IIBs) showed a prognostic relevance in patients with metastatic CRC (mCRC). We aimed at evaluating the prognostic power of a new comprehensive biomarker, the Pan-Immune-Inflammation Value (PIV), in patients with mCRC receiving first-line therapy.

**Methods:**

In the present pooled-analysis, we included patients enrolled in the *Valentino* and TRIBE trials. PIV was calculated as: (neutrophil count × platelet count × monocyte count)/lymphocyte count. A cut-off was determined using the maximally selected rank statistics method. Generalised boosted regression (GBR), the Kaplan–Meier method and Cox hazards regression models were used for survival analyses.

**Results:**

A total of 438 patients were included. Overall, 208 patients (47%) had a low-baseline PIV and 230 (53%) had a high-baseline PIV. Patients with high PIV experienced a worse PFS (HR, 1.66; 95% CI, 1.36–2.03, *P* < 0.001) and worse OS (HR, 2.01; 95% CI, 1.57–2.57; *P* < 0.001) compared to patients with low PIV. PIV outperformed the other IIBs in the GBR model and in the multivariable models.

**Conclusion:**

PIV is a strong predictor of survival outcomes with better performance than other well-known IIBs in patients with mCRC treated with first-line therapy. PIV should be prospectively validated to better stratify mCRC patients undergoing first-line therapy.

## Background

Even in the era of molecular selection,^1^ a non-negligible fraction of patients with metastatic colorectal cancer (mCRC) receiving first-line treatment has poor outcomes.^2^ Thus, the identification of new biomarkers for a better prognostic stratification and prediction of treatment outcomes is mandatory. Most of the biomarkers investigated so far are tumour-related, with less focus on host-related factors. Inflammation and immunity play a fundamental role in colorectal cancer initiation and progression,^3,4^ and several blood-based, easy-to-obtain, immune-inflammatory biomarkers (IIBs) have been investigated in cancer patients.^5^ Among others, neutrophil-to-lymphocyte ratio (NLR), platelets and monocytes showed a prognostic relevance in the advanced setting,^6–8^ but the clinical usefulness of such single biomarkers is limited by their low discriminative ability. Since the interplay between immunity, inflammation and cancer relies on complex networks, the use of a composite biomarker encompassing different immune-inflammatory populations and reflecting the global inflammation status could achieve a more robust prognostic power. Of note, the systemic immune-inflammation index (SII) based on lymphocyte, neutrophil and platelet counts, but not monocytes, was first developed for prognostic stratification in patients with hepatocellular carcinoma^9^ and demonstrated a certain relevance also in mCRC.^10^

In patients with mCRC, the use of such IIBs should be assessed in large datasets of patients enrolled in modern trials. In the present pooled-analysis of patients with mCRC receiving first-line therapy in the frame of two randomised academic trials, *Valentino* and TRIBE, we aimed to evaluate the prognostic power of a new biomarker, the Pan-Immune-Inflammation Value (PIV), including all the immune-inflammatory populations from peripheral blood with a proved prognostic relevance in mCRC.

## Methods

### Patients population

The *Valentino* study (NCT02476045) was a multicentre, randomised, open-label Phase 2 trial that enrolled 229 patients and showed that, in patients with *RAS* wild-type mCRC, panitumumab plus FOLFOX-4 induction followed by maintenance therapy with single-agent panitumumab (arm B) achieved inferior PFS compared to the same induction regimen followed by panitumumab plus 5-FU/LV (arm A).^11^ The TRIBE study (NCT00719797) was a multicentre, randomised, open-label Phase 3 trial by Gruppo Oncologico del Nord Ovest (GONO) that enrolled 508 patients and showed that, in patients with molecularly unselected mCRC, first-line FOLFOXIRI plus bevacizumab achieved superior PFS and OS compared with FOLFIRI plus bevacizumab.^12^

For the present study, we selected patients enrolled in the two trials with available *RAS* and *BRAF* mutational status, complete baseline blood-cell count at cycle 1, day 1 (prior to first treatment cycle administration) and clinicopathological data including but not limited to prior exposure to adjuvant chemotherapy, primary tumour resection and primary tumour sidedness.

### Statistical analyses

In order to represent a weight of the interaction between inflammatory pro-tumour populations (i.e. neutrophils, platelets and monocytes) and anti-cancer immune populations (i.e. lymphocytes), PIV was calculated as: [neutrophil count (10^3^/mmc) × platelet count (10^3^/mmc) × monocyte count (10^3^/mmc)]/lymphocyte count (10^3^/mmc). Maximally selected rank statistics method for PFS was used to find an optimal cut-off value^13^ to stratify patients in low PIV vs high PIV. NLR was calculated as: neutrophil count (10^3^/mmc)/lymphocyte count (10^3^/mmc). NLR was defined high if >3, platelet count was defined high if >310 × 10^3^/mmc and monocyte count was defined high if >0.5 × 10^3^/mmc based on literature data.^6–8^ SII was calculated as [neutrophil count (10^3^/mmc) × platelet count(10^3^/mmc)/lymphocyte count (10^3^/mmc) and defined high if >730 based on literature data.^10^

Fisher exact test, Chi-square test, Mann–Whitney U test or Kruskal-Wallis test, as appropriate, were used to analyse the association between baseline PIV and the other clinicopathological characteristics. PFS was defined as the time from randomisation to disease progression or death from any cause. OS was defined as the time from randomisation to death from any cause. Generalised boosted regression was used to screen the association of PIV and the other IIBs with PFS and OS.^14,15^ Further survival analyses were performed using the Kaplan–Meier method and the Cox proportional hazards regression models. All the variables showing a *P* below the significance threshold in the univariate models were included in a multivariable model. The variables showing a *P* below the significance threshold in the multivariable models were considered to be independent prognostic factors. All tests were 2-sided with a significance threshold of 0.05. Statistical analyses were performed using the R (version 3.5.0) and R Studio (version 1.1.447).

## Results

### Patients characteristics according to Pan-Immune-Inflammation Value

A total of 438 patients were included in the present analysis: 207 from the *Valentino* study and 231 from the TRIBE study. The process of patients’ selection is illustrated in Supplementary Fig. S1. In terms of patients’ characteristics, the subsets of patients included in the present study was representative of the overall trial populations (Supplementary Table S1). Median PIV in the entire study population was 417 (IQR, 239–780). The distribution of median PIV according to patients’ and disease characteristics is shown in Supplementary Table S2.

The optimal cut-off value for PIV using a maximally selected rank statistics method for PFS was 390 (Supplementary Fig. S2). Overall, 208 patients (47%) had a low PIV and 230 (53%) had a high PIV. The distribution of high vs low PIV patients in the two studies was well balanced (Table 1). Compared to patients with low PIV, a higher proportion of patients with high PIV had ECOG PS1 (*P* < 0.001), no primary tumour resection (*P* < 0.001), presence of synchronous metastases (*P* = 0.003) and more than 1 site of metastases (*P* = 0.032) (Table 1). The association between PIV and the classical immune-inflammatory biomarkers is shown in Supplementary Table S3.

**Table 1:** Pan-Immune-Inflammation Value (PIV) according to patients’ and disease baseline characteristics.

Characteristics	Total (*N* = 438) *N* (%)	PIV low (*N* = 208) *N* (%)	PIV high (*N* = 230) *N* (%)	P*
Age (years)				0.111
Median	62	62	60	
IQR	53–68	55–68	52–67	
Gender				0.608
Female	163 (37)	80 (38)	83 (36)	
Male	275 (63)	128 (62)	147 (64)	
ECOG PS				**<0.001**
0	356 (81)	186 (89)	170 (74)	
1	82 (19)	22 (11)	60 (26)	
Prior adjuvant treatment				0.127
No	376 (86)	173 (83)	203 (88)	
Yes	62 (14)	35 (17)	27 (12)	
Primary tumour resected				**<0.001**
No	133 (30)	45 (22)	88 (38)	
Yes	305 (70)	163 (78)	142 (62)	
Liver-limited disease				0.066
No	307 (70)	137 (66)	170 (74)	
Yes	131 (30)	71 (34)	60 (26)	
Synchronous metastases				**0.003**
No	97 (22)	59 (28)	38 (17)	
Yes	341 (78)	149 (72)	192 (83)	
Number of metastatic sites				**0.032**
1	181 (41)	97 (47)	84 (37)	
>1	257 (59)	111 (53)	146 (63)	
Primary tumour sidedness				0.240
Left	330 (75)	162 (78)	168 (73)	
Right	108 (25)	46 (22)	62 (27)	
*RAS/BRAF* status				0.514
* RAS/BRAF* wild-type	276 (63)	127 (61)	149 (65)	
* RAS* mut	146 (33)	75 (36)	71 (31)	
* BRAF* mut	16 (4)	6 (3)	10 (4)	
Study				0.659
* Valentino*	207 (47)	112 (54)	119 (52)	
TRIBE	231 (53)	96 (46)	111 (48)	
Chemotherapy backbone				0.324
Doublet	321 (73)	157 (75)	164 (71)	
Triplet	117 (27)	51 (25)	66 (29)	

*IQR* interquartile range, *ECOG* Eastern Cooperative Oncology Group, *PS* performance status, *PIV* Pan-Immune-Inflammation Value.

*Fisher exact test, Chi-square test, Mann–Whitney test or Kruskal-Wallis test as appropriate.

*P* below the significance threshold are reported in bold.

### Prognostic analyses according to Pan-Immune-Inflammation Value

**Fig. 1:**
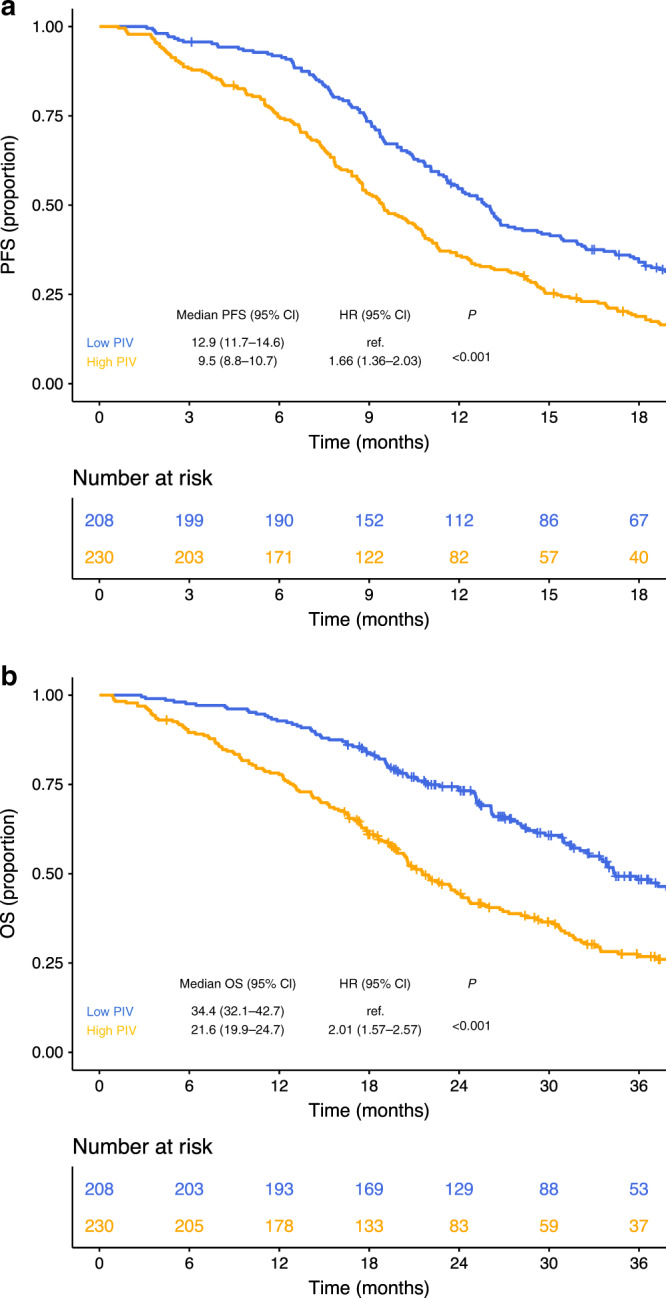
Kaplan–Meier curves for PFS (**a**) and OS (**b**) in the overall population according to PIV. Blue lines indicate patients with low PIV whereas yellow lines indicate patients with high PIV. Patients with high PIV had worse survival outcomes compared to patients with low PIV.

**Fig. 2:**
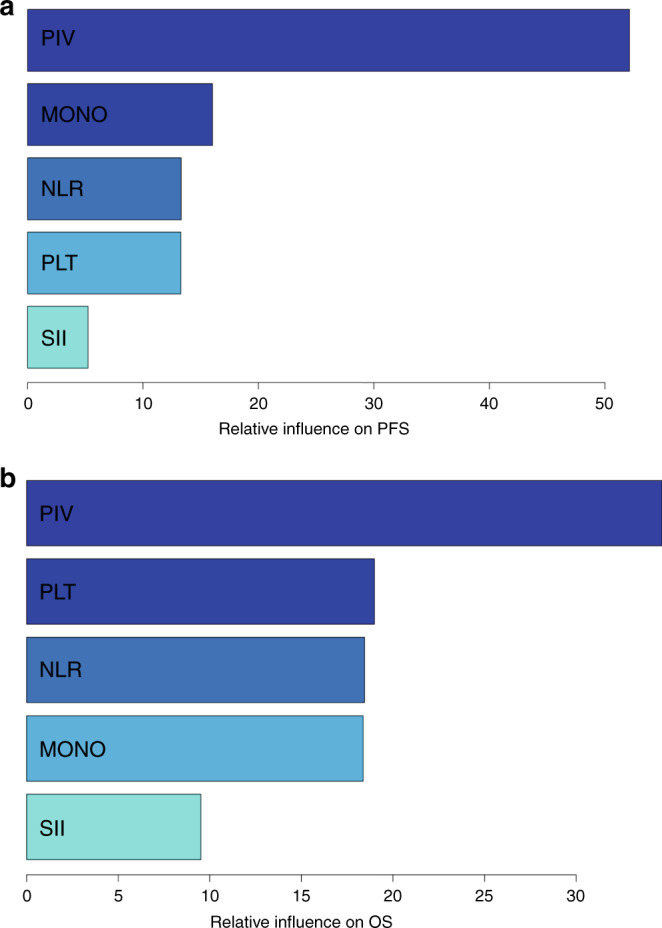
Bar graph showing the relative influence by generalised boosted regression on PFS (**a**) and OS (**b**) of the immune-inflammatory biomarkers analysed. PIV showed the highest relative influence among the biomarkers analysed.

Median follow-up was 38.4 months (IQR, 27.4–50.9). A total of 389 PFS events were recorded with a pooled median PFS of 11.1 months (95% CI, 10.3–11.9). Median PFS was 9.5 months (95% CI, 8.8–10.7) for patients with high PIV and 12.9 months (95% CI, 11.7–14.6) for those with low PIV (HR high vs low, 1.66; 95% CI, 1.36–2.03, *P* < 0.001) (Fig. 1, panel a). Similar results were observed in the two separate populations in the *Valentino* and TRIBE studies (Supplementary Fig. S3, panels a and b, respectively). At univariate analysis, also NLR, platelet count, monocyte count and SII were significantly associated with PFS (Table 2). In the generalised boosted regression model, PIV showed the higher relative influence on PFS among the IIBs (Fig. 2, panel a). In the multivariable model including all the variables significantly associated with PFS, PIV was the only IIB that showed an independent prognostic impact on PFS (adjusted HR high vs low, 1.53; 95% CI, 1.09–2.15; *P* = 0.015) (Table 2).

**Table 2:** Cox proportional hazards regression models for PFS.

Characteristics	Univariate analysis HR (95% CI)	*P*	Multivariable model HR (95% CI)	*P*
Age (years)^a^		0.291		–
53	Ref		–	
68	0.92 (0.80–1.07)		–	
Gender		0.865		–
Female	Ref		–	
Male	0.98 (0.80–1.21)		–	
ECOG PS		**0.001**		**0.002**
0	Ref		Ref	
1	1.53 (1.19–1.97)		1.53 (1.17–1.99)	
Prior adjuvant treatment		**0.025**		0.580
No	Ref		Ref	
Yes	0.72 (0.54–0.96)		1.12 (0.75–1.68)	
Primary tumour resected		**0.009**		0.392
No	Ref		Ref	
Yes	0.75 (0.60–0.93)		0.90 (0.71–1.15)	
Liver-limited disease		0.137		–
No	Ref		–	
Yes	0.85 (0.68–1.05)		–	
Synchronous metastases		**<0.001**		**0.036**
No	Ref		Ref	
Yes	1.55 (1.21–1.98)		1.46 (1.03–2.09)	
Number of metastatic sites		**<0.001**		**<0.001**
1	Ref		Ref	
>1	1.48 (1.21–1.82)		1.49 (1.19–1.85)	
Primary tumour sidedness		**0.005**		0.084
Left	Ref		Ref	
Right	1.39 (1.11–1.74)		1.24 (0.97–1.59)	
*RAS/BRAF* status		**0.001**		**0.015**
*RAS/BRAF* wt	Ref		Ref	
*RAS* mut	1.18 (0.96–1.46)		1.07 (0.85–1.34)	
*BRAF* mut	2.46 (1.48–4.08)		2.37 (1.40–4.03)	
Study		0.784		**–**
* Valentino*	Ref		–	
TRIBE	0.97 (0.79–1.19)		–	
Backbone		0.485		**–**
Doublet	Ref		–	
Triplet	0.92 (0.74–1.15)		–	
NLR		**0.025**		0.462
Low	Ref		Ref	
High	1.26 (1.03–1.54)		0.89 (0.66–1.21)	
PLT		**<0.001**		0.509
Low	Ref		Ref	
High	1.44 (1.18–1.77)		1.09 (0.84–1.41)	
MONO		**0.001**		0.885
Low	Ref		Ref	
High	1.40 (1.14–1.71)		0.98 (0.76–1.26)	
SII		**0.003**		0.871
Low	Ref		Ref	
High	1.36 (1.11–1.66)		0.97 (0.68–1.38)	
PIV		**<0.001**		**0.015**
Low	Ref		Ref	
High	1.66 (1.36–2.03)		1.53 (1.09–2.15)	

*HR* hazard ratio, *CI* confidence interval, *ECOG* Eastern Cooperative Oncology Group, *PS* performance status, *Ref* reference, *NLR* neutrophil-to-lymphocyte ratio, *PLT* platelet count, *MONO* monocyte count, *SII* systemic immune-inflammation index, *PIV* Pan-Immune-Inflammation Value.

^a^The two values represent the first and third quartile, respectively, of the variable distribution.

A total of 269 OS events were reported during the study period with a pooled median OS of 28.5 months (95% CI, 25.6–31.61). Median OS was 21.6 months (95% CI, 19.9–24.7) for patients with high PIV and 34.4 months (95% CI, 32.1–42.7) for patients with low PIV (HR high vs low, 2.01; 95% CI, 1.57–2.57; *P* < 0.001) (Fig. 1, panel b). Results were consistent when the two populations were analysed separately (Supplementary Fig. S4). NLR, platelet count, monocyte count and SII were also significantly associated with OS (Table 3). In the generalised boosted regression model, PIV showed the higher relative influence on OS among the immune-inflammatory biomarkers (Fig. 2, panel b). As for PFS, PIV was the only inflammation-based biomarker that showed an independent prognostic impact on OS (adjusted HR high vs low, 1.55; 95% CI, 1.02–2.37, *P* = 0.042) (Table 3).

**Table 3:** Cox proportional hazard regression models for OS.

Characteristics	Univariate analysis HR (95% CI)	*P*	Multivariable model HR (95% CI)	*P*
Age (years)^a^		0.383		–
53	Ref		–	
68	1.08 (0.91–1.29)		–	
Gender		0.549		–
Female	Ref			
Male	0.93 (0.73–1.19)		–	
ECOG PS		**<0.001**		**<0.001**
0	Ref		Ref	
1	1.96 (1.47–2.63)		2.08 (1.53–2.85)	
Prior adjuvant treatment		**0.002**		0.998
No	Ref		Ref	
Yes	0.56 (0.38–0.81)		1.00 (0.57–1.75)	
Primary tumour resected		**0.014**		0.391
No	Ref		Ref	
Yes	0.72 (0.56–0.94)		0.88 (0.66–1.18)	
Liver-limited disease		0.151		–
No	Ref		–	
Yes	0.82 (0.63–1.07)		–	
Synchronous metastases		**<0.001**		**0.043**
No	Ref		Ref	
Yes	1.87 (1.36–2.57)		1.63 (1.02–2.61)	
Number of metastatic sites		**0.001**		**0.009**
1	Ref		Ref	
>1	1.51 (1.17–1.95)		1.43(1.09–1.88)	
Primary tumour sidedness		**0.001**		**0.014**
Left	Ref		Ref	
Right	1.56 (1.20–2.03)		1.42 (1.08–1.88)	
*RAS*/*BRAF* status		**<0.001**		**0.003**
*RAS/BRAF* wt	Ref		Ref	
*RAS* mut	1.36 (1.06–1.74)		1.29 (0.99–1.69)	
*BRAF* mut	2.88 (1.72–4.84)		2.65 (1.55–4.54)	
Study		0.497		**–**
* Valentino*	Ref		–	
TRIBE	0.91 (0.70–1.19)		–	
Backbone		0.789		**–**
Doublet	Ref		–	
Triplet	1.04 (0.80–1.34)		–	
NLR		**<0.001**		0.402
Low	Ref		Ref	
High	1.56 (1.23–1.99)		1.17 (0.81–1.68)	
PLT		**<0.001**		0.168
Low	Ref		Ref	
High	1.67 (1.31–2.12)		1.23 (0.92–1.64)	
MONO		**0.001**		0.691
Low	Ref		Ref	
High	1.52 (1.19–1.94)		0.94 (0.70–1.27)	
SII		**<0.001**		0.677
Low	Ref		Ref	
High	1.58 (1.24–2.01)		0.91 (0.59–1.41)	
PIV		**<0.001**		**0.042**
Low	Ref		Ref	
High	2.01 (1.57–2.57)		1.55 (1.02–2.37)	

*HR* hazard ratio, *CI* confidence interval, *ECOG* Eastern Cooperative Oncology Group, *PS* performance status, *Ref* reference, *NLR* neutrophil-to-lymphocyte ratio, *PLT* platelet count, *MONO* monocyte count, *SII* systemic immune-inflammation index, *PIV* Pan-Immune-Inflammation Value.

^a^The two values represent the first and third quartile, respectively, of the variable distribution.

### Predictive analyses according to Pan-Immune-Inflammation Value

**Fig. 3:**
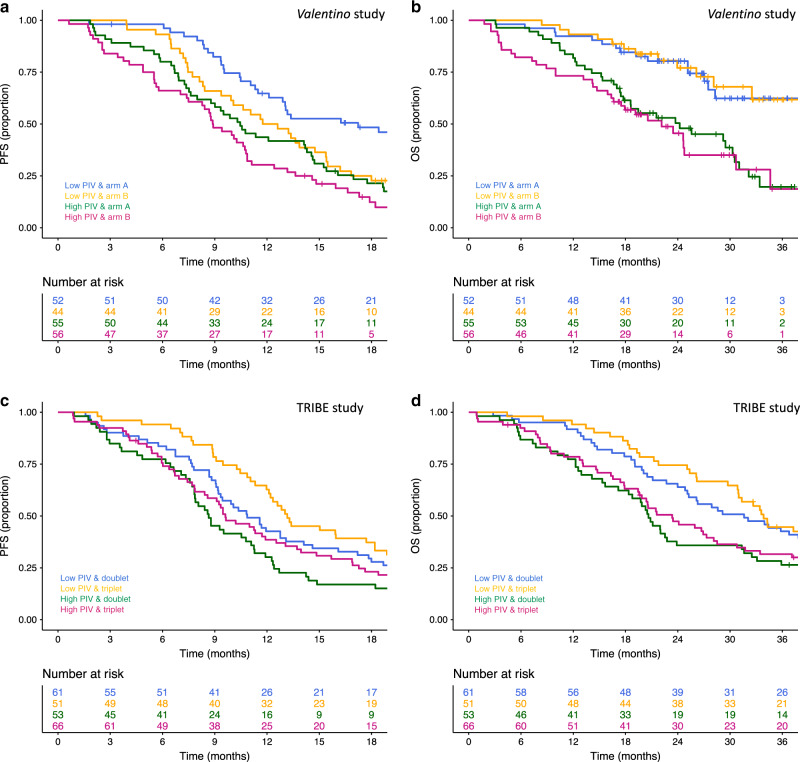
Kaplan–Meier curve for PFS and OS according to PIV and treatment arm in the *Valentino* study (**a** and **b**, respectively) and in the TRIBE study (**c** and **d**, respectively). PIV was not significantly associated with a differential effect of the two maintenance arms in the *Valentino* study nor with a differential effect of triplet-based vs doublet-based therapy in the TRIBE study.

In the *Valentino* study, PIV was not significantly associated with a differential effect of the two maintenance arms in terms of PFS (interaction *P* = 0.449) and OS (interaction *P* = 0.612) (Fig. 3, panels a and b, respectively).

Similar results were observed about the predictive role of PIV in the TRIBE study for patients treated with triplet-based vs doublet-based therapy (interaction *P* for PFS = 0.924; interaction *P* for OS = 0.951) (Fig. 3, panels C and D). Supplementary Table S4 summarises the results of the predictive analyses.

## Discussion

In the present pooled-analysis of two first-line trials, we investigated PIV as a new inflammation-based biomarker that integrates NLR, platelet count and monocyte count. PIV demonstrated an extensive and powerful prognostic impact on both PFS and OS in patients with mCRC receiving first-line chemotherapy plus a biological agent. We observed that patients with high baseline PIV had significantly worse survival outcomes compared to patients with low baseline PIV. PIV had the highest relative influence on survival outcomes in the generalised boosted regression models including the other canonical IIBs (i.e. NLR, platelet count, monocyte count) and was the only one that retained an independent prognostic role for PFS and OS in the multivariable models.

To avoid a fragmentated information about systemic inflammation, both nomogram systems and scores have been also developed to integrate the various components in the prognostic modelling of CRC,^7,16^ but there is no consensus about the best approach. Rather than analysing the individual contribution of each cellular components (i.e. lymphocytes, neutrophils, platelets and monocytes) on clinical outcomes and then building a calculator (i.e. nomogram or score) in a statistical-driven approach, we tested the prognostic relevance of a new biomarker incorporating lymphocytes, neutrophils, platelets and monocytes in a way that allowed us to “globally quantify” the cellular compartment of systemic inflammation (i.e. biological-driven approach).

Of note, PIV also outperformed another multicomponent inflammatory index, the SII that does not include information about monocyte count.

Among circulating white blood cells, monocytes are one of the most important subpopulations with an emerging role in cancer progression^17^ and potential prognostic impact also in patients with mCRC.^7^ Indeed, circulating macrophages represent the primary source of tumour-associated macrophages (TAMs) and the peripheral monocyte count was associated with the density of TAMs in colorectal cancer.^18^ Peripheral monocytes are also the source of monocytic (M-) myeloid-derived suppressor cells (MDSCs) that, together with polymorphonuclear (PMN-) MDSCs, characterise a population of immune cells driving immunosuppression and progression in mCRC.^19,20^ Of note, PMN-MDSCs are a particular phenotype of circulating neutrophils,^21^ so using a biomarker like PIV including monocytes and neutrophils rather than neutrophils only might easily summarise the immunosuppressive contribution of the two components of MDSCs without the need of complex cytofluorimetric analyses.

Even if with some limitations consisting in its retrospective nature and lack of prospective validation, our study included patients enrolled in two randomised clinical trials guaranteeing a high quality of data, especially in weighting the prognostic contribution of monocyte count, a parameter usually not included in the case report forms of clinical studies,^22^ particularly academic ones.

In conclusion, our study identifies PIV as a new IIB strongly associated with overall prognosis of mCRC patients receiving first-line treatment and outperforms the other well-known IIBs, suggesting its possible role as a stratification factor in future first-line clinical trials. Further studies should assess the role of PIV as predictive biomarker, particularly regarding its early modifications during treatment as a potentially dynamic biomarker associated with treatment outcomes, and in different settings (for instance, patients with pre-treated mCRC or early stage) or histologies, and with specific regard to immunotherapy approaches.

## Supplementary information

Supplementary Information

## Data Availability

The data that support the findings of this study are available from the corresponding author upon reasonable request.
