# Supplementary material for: The Pan-Immune-Inflammation Value is a new prognostic biomarker in metastatic colorectal cancer: results from a pooled-analysis of the Valentino and TRIBE first-line trials
Source: Br J Cancer. 2020 May 19;123(3):403–9. doi: 10.1038/s41416-020-0894-7 (PMC7403416; doi:10.1038/s41416-020-0894-7)

*Article*

**The Pan-Immune-Inflammation Value is a new prognostic biomarker in metastatic colorectal cancer: results from a pooled-analysis of the *Valentino* and TRIBE first-line trials**

Running title: *Pan-Immune-Inflammation Value in colorectal cancer*

## SUPPLEMENTARY INFORMATION

**Supplementary Table S1. Patients' characteristics in the pooled population and in the separate subgroups of patients included from the *Valentino* study and the TRIBE study.**

**Supplementary Table S2. Pan-immune-Inflammation Value (PIV) according to patients' and disease baseline characteristics.**

**Supplementary Table S3. Association between PIV and classic immune-inflammatory biomarkers.**

**Supplementary Table S4. Predictive analyses according to PIV in the *Valentino* and TRIBE studies.**

**Supplementary Figure S1. Flow chart showing the process of patients' selection.**

**Supplementary Figure S2. Dot plot showing the standardized log-rank test statistic for PFS according to PIV value.**

**Supplementary Figure S3. Kaplan-Meier curve for PFS according to PIV in the Valentino (panel A) and TRIBE (panel B) study populations.**

**Supplementary Figure S4. Kaplan-Meier curve for OS according to PIV in the Valentino (panel A) and TRIBE (panel B) study populations.**

**Supplementary Table S1. Patients' characteristics in the pooled population and in the separate subgroups of patients included from the *Valentino* study and the TRIBE study.**

| Characteristics | Pooled population (*N*=438) *N* (%) | *Valentino* study (*N*=207) *N* (%) | TRIBE study (*N*=231) *N (%)* |
|---|---|---|---|
| **Age** (years) | | | |
| Median | 62 | 62 | 60 |
| IQR | 53-68 | 54-69 | 53-67 |
| **Gender** | | | |
| Female | 163 (37) | 67 (32) | 96 (42) |
| Male | 275 (63) | 140 (68) | 135 (58) |
| **ECOG PS** | | | |
| 0 | 356 (81) | 153 (74) | 203 (88) |
| 1 | 82 (19) | 54 (26) | 28 (12) |
| **Prior adjuvant treatment** | | | |
| No | 376 (86) | 176 (85) | 200 (87) |
| Yes | 62 (14) | 31 (15) | 31 (13) |
| **Primary tumour resected** | | | |
| No | 133 (30) | 55 (27) | 56 (24) |
| Yes | 305 (70) | 130 (63) | 175 (76) |
| **Liver-limited disease** | | | |
| No | 307 (70) | 132 (64) | 175 (76) |
| Yes | 131 (30) | 75 (36) | 56 (24) |
| **Synchronous metastases** | | | |
| No | 97 (22) | 44 (21) | 53 (23) |
| Yes | 341 (78) | 163 (79) | 178 (77) |
| **Number of metastatic sites** | | | |
| 1 | 181 (41) | 117 (57) | 64 (28) |
| > 1 | 257 (59) | 90 (43) | 167 (72) |
| **Primary tumour sidedness** | | | |
| Left | 330 (75) | 178 (86) | 152 (66) |
| Right | 108 (25) | 29 (14) | 79 (34) |
| ***RAS/BRAF* status** | | | |
| *RAS/BRAF* wild-type | 276 (63) | 207 (100) | 69 (30) |
| *RAS* mutated | 146 (33) | 0 (0) | 146 (63) |
| *BRAF* mutated | 16 (4) | 0 (0) | 16 (7) |

*Abbreviations.* ECOG: Eastern Cooperative Oncology Group; PS: performance status.

**Supplementary Table S2. Pan-immune-Inflammation Value (PIV) according to patients' and disease baseline characteristics.**

| Characteristics | PIV Median (IQR) | *P** |
|---|---|---|
| **Gender** | | 0.666 |
| Female (*N*=163) | 396 (230-800) | |
| Male ( *N* =275) | 418 (250-754) | |
| **ECOG PS** | | **<0.001** |
| 0 (*N*=356) | 368 (228-700) | |
| 1 (*N*=82) | 568 (377-1578) | |
| **Prior adjuvant treatment** | | **0.032** |
| No (*N*=376) | 426 (261-794) | |
| Yes (*N*=62) | 340 (181-606) | |
| **Primary tumour resected** | | **<0.001** |
| No (*N*=133) | 557 (292-1005) | |
| Yes (*N*=305) | 364 (221-679) | |
| **Liver-limited disease** | | **0.012** |
| No (*N*=307) | 428 (262-856) | |
| Yes (*N*=131) | 341 (198-635) | |
| **Synchronous metastases** | | **0.001** |
| No (*N*=97) | 317 (211-522) | |
| Yes (*N*=341) | 442 (262-835) | |
| **Number of metastatic sites** | | **0.002** |
| 1 (*N*=181) | 347 (212-623) | |
| > 1 (*N*=257) | 441 (273-1005) | |
| **Primary tumour sidedness** | | 0.295 |
| Left (*N*=330) | 398 (234-773) | |
| Right (*N*=108) | 452 (270-828) | |
| ***RAS/BRAF* status** | | 0.395 |
| *RAS/BRAF* wild-type (*N*=276) | 420 (245-814) | |
| *RAS* mutated (*N*=146) | 376 (226-740) | |
| *BRAF* mutated (*N*=16) | 602 (268-1056) | |
| **Study** | | 0.565 |
| *Valentino* (*N*=207) | 421 (252 -814) | |
| TRIBE (*N*=231) | 400 (237-774) | |
| **Chemotherapy Backbone** | | 0.838 |
| Doublet (*N*=321) | 415 (239-796) | |
| Triplet (*N*=117) | 429 (248-748) | |

* Mann–Whitney test or Kruskal-Wallis test, as appropriate

*Abbreviations.* IQR: interquartile range; ECOG: Eastern Cooperative Oncology Group; PS: performance status; PIV: Pan-immune Inflammation Value.

**Supplementary Table S3. Association between PIV and classic immune-inflammatory biomarkers.**

| Characteristics | Total (*N*=438) *N* (%) | PIV low (*N*=208) *N* (%) | PIV high (*N*=230) *N* (%) | *P** |
|---|---|---|---|---|
| **NLR** | | | | **<0.001** |
| Low | 251 (57) | 177 (85) | 74 (32) | |
| High | 187 (43) | 31 (15) | 156 (68) | |
| **PLT** | | | | **<0.001** |
| Low | 264 (60) | 177 (85) | 87 (38) | |
| High | 174 (40) | 31 (15) | 143 (62) | |
| **MONO** | | | | |
| Low | 193 (44) | 147 (71) | 46 (20) | **<0.001** |
| High | 245 (56) | 61 (29) | 184 (80) | |
| **SII** | | | | |
| Low | 206 (47) | 173 (83) | 33 (14) | **<0.001** |
| High | 232 (53) | 35 (17) | 197 (86) | |

* Fisher exact test or Chi square test as appropriate

*Abbreviations.* IQR: interquartile range; PIV:  Pan-immune-Inflammation Value; NLR: neutrophil-to-lymphocyte ration; PLR: platelet count; MONO: monocyte count; SII: systemic immune-inflammation index.

**Supplementary Table S4. Predictive analyses according to PIV in the *Valentino* and TRIBE studies.**

| *Valentino* study | | | | | | | |
|---|---|---|---|---|---|---|---|
| **Outcomes** | **Low PIV** | | | **High PIV** | | | **Interaction test *P*** |
| | **Arm A** | **Arm B** | **HR (95% CI)** | **Arm A** | **Arm B** | **HR (95% CI)** | |
| Median PFS, months (95% CI) | 17.2 (12.9-22.9) | 12.2 (9.9-15.4) | 1.61 (1.01-2.58) | 10.5 (8.8-14.6) | 8.9 (7.5-10.9) | 1.26 (0.85-1.86) | 0.449 |
| Median OS, months (95% CI) | NA (28.3-NA) | NA (32.5-NA) | 0.94 (0.44-2.00) | 24.2 (17.8-31.1) | 22.1 (16.6-NA) | 1.15 (0.71-1.86) | 0.612 |
| **TRIBE study** | | | | | | | |
| **Outcomes** | **Low PIV** | | | **High PIV** | | | **Interaction test *P*** |
| | **Doublet** | **Triplet** | **HR (95% CI)** | **Doublet** | **Triplet** | **HR (95% CI)** | |
| Median PFS, months (95% CI) | 10.9 (9.2-14.6) | 13.2 (12.1-18.2) | 0.83 (0.56-1.23) | 8.7 (7.8-11.3) | 9.6 (8.4-13.1) | 0.83 (0.57-1.20) | 0.924 |
| Median OS, months (95% CI) | 31.6 (25.1-42.7) | 33.9 (31.0-44.2) | 0.93 (0.59-1.45) | 20.6 (18.4-31.4) | 23.4 (19.6-30.8) | 0.94 (0.63-1.41) | 0.951 |

*Abbreviations:* PIV: Pan-immune-Inflammation Value; PFS: progression-free survival; OS: overall survival; HR: hazard ratio.

**Supplementary Figure S1. Flow chart showing the process of patients' selection.**

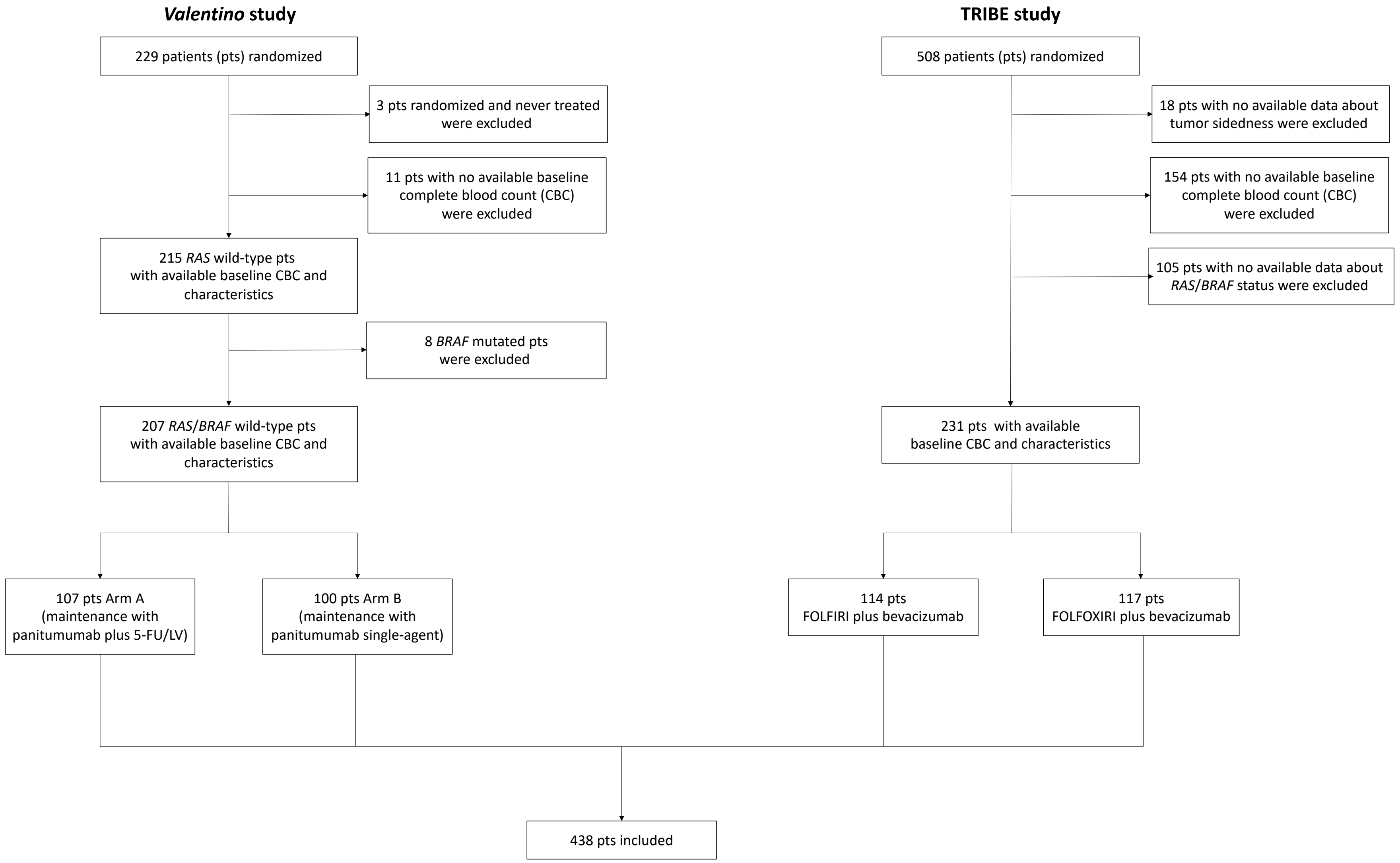

**Supplementary Figure S2. Dot plot showing the standardized log-rank test statistic for PFS according to PIV value.** The best cut-off value for PIV was 390, with a standardized log-rank test statistic (M) of 4.932.

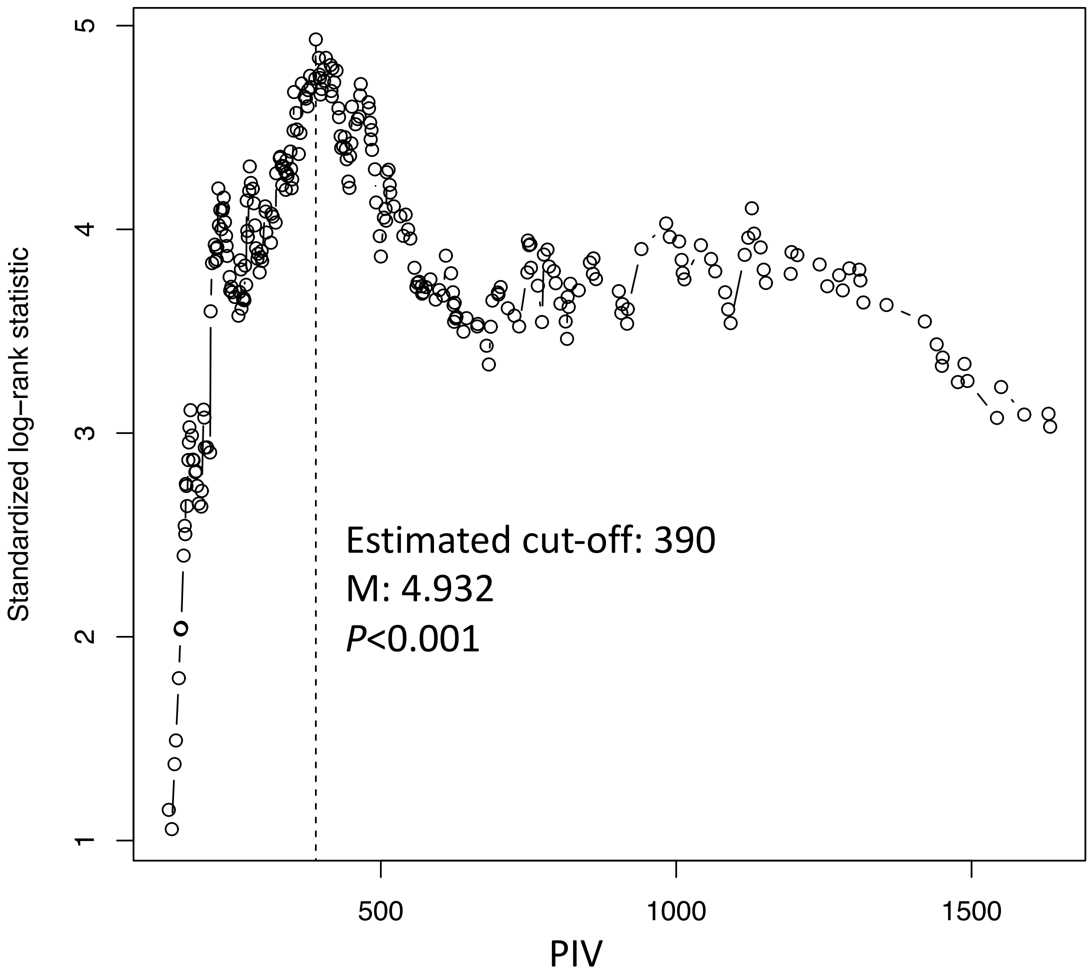

**Supplementary Figure S3. Kaplan-Meier curve for PFS according to PIV in the Valentino (panel A) and TRIBE (panel B) study populations.** Blue lines indicate patients with low PIV whereas yellow lines indicate patients with high PIV. Patients with high PIV had worse PFS respect to patients with low PIV regardless of the study.

A

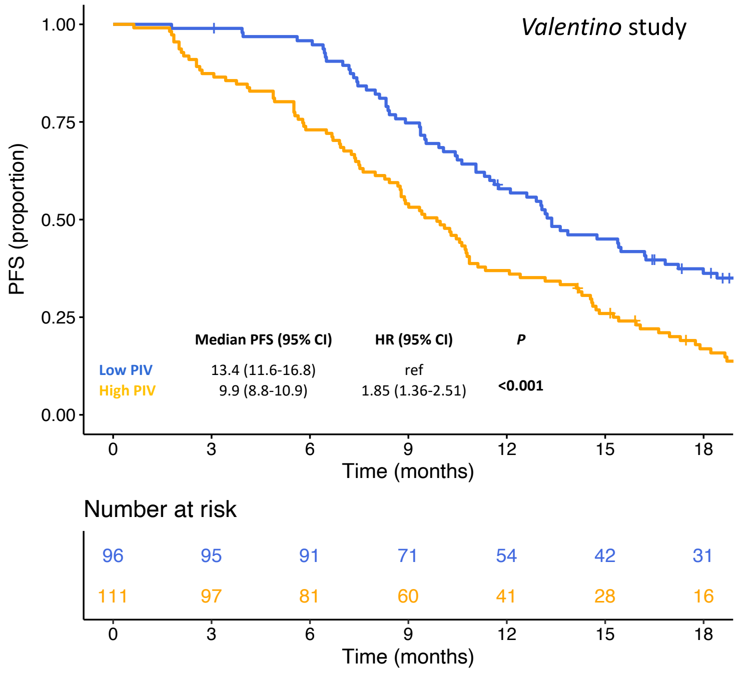


B

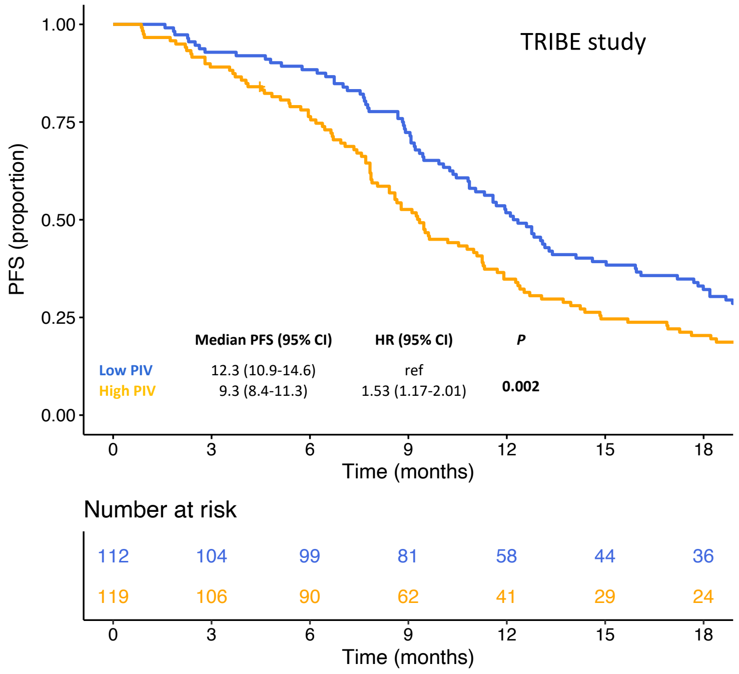

**Supplementary Figure S4. Kaplan-Meier curve for OS according to PIV in the Valentino (panel A) and TRIBE (panel B) study populations.** Blue lines indicate patients with low PIV whereas yellow lines indicate patients with high PIV. Patients with high PIV had worse OS respect to patients with low PIV regardless of the study.

A

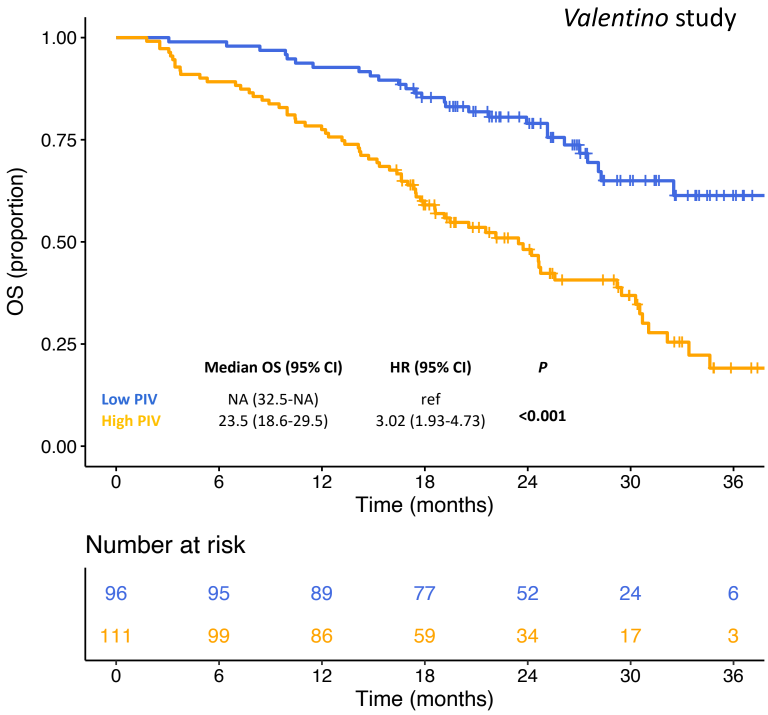


B

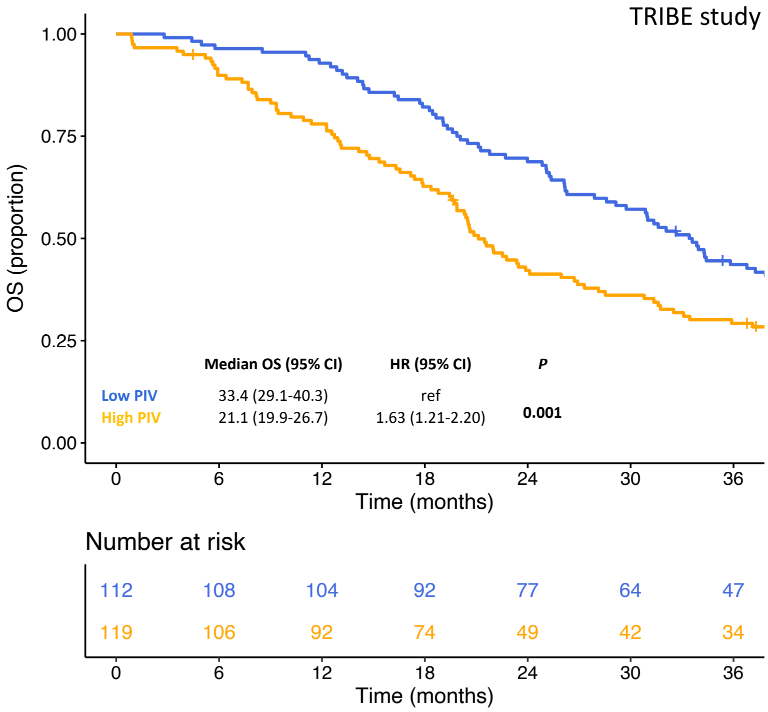

Supplement: Supplementary file 1 — Supplementary Information [file 41416_2020_894_MOESM1_ESM.pdf]
